# Keepin’ in De Middle Ob De Road

**Published:** 1909-07

**Authors:** Victoria A. Hardy Duggan


### Keepin' in de Middle ob de Road.

My back is weak an' my staff is slim,
My hat's all holes an' my eyes is dim;
But I totes my pack, an' neber looks back,
Kase I keeps in de middle ob de road.

De road am rough an' it's sure mighty hard
Ter keep my feet a-movin' in de way ob de Lord,
But my heart is light, kase I tries ter do right
By a-keepin' in the middle ob de road.

Ol' Satan lopes on, clos't at my back,
An' he ax for ter ride upon my pack;
But I keeps him off, wid a sneer an' a scoff,
An' a-keepin' in de middle ob de road.

I trabbles some slow, an' I trabbles some fast,
So I can git home by de trumpets' blast;
Kase rest to my feet will sure be sweet,
So I'se keepin' in de middle ob de road.

VICTORIA A. HARDY DUGGAN.

